# Inclusion of sorghum, millet and cottonseed meal in broiler diets: a meta-analysis of effects on performance

**DOI:** 10.1017/S1751731115000282

**Published:** 2015-03-04

**Authors:** D. I. Batonon-Alavo, M. Umar Faruk, P. Lescoat, G. M. Weber, D. Bastianelli

**Affiliations:** 1 INRA, UR83 Recherches Avicoles, F-37380 Nouzilly, France; 2 Research Centre for Animal Nutrition and Health, DSM Nutritional Products France, F-68128 Village-Neuf, France; 3 AgroParisTech, UMR 1048 SADAPT, F-75005 Paris, France; 4 DSM Nutritional Products Ltd, Nutrition Innovation Center, CH-4002 Basel, Switzerland; 5 CIRAD, UMR SELMET, Systèmes d'élevage méditerranéens et tropicaux, Baillarguet TA C-112/A, F-34398 Montpellier, France

**Keywords:** meta-analysis, broiler, sorghum, millet, cottonseed meal

## Abstract

A meta-analysis was conducted (i) to evaluate broiler response to partial or total substitution of corn by sorghum and millet and (ii) to determine the effect of soybean meal replacement by cottonseed meal in broiler diet. The database included 190 treatments from 29 experiments published from 1990 to 2013. Bird responses to an experimental diet were calculated relative to the control (*Experimental−Control*), and were submitted to mixed-effect models. Results showed that diets containing millet led to similar performance as the corn-based ones for all parameters, whereas sorghum-based diets decreased growth performance. No major effect of the level of substitution was observed with millet or cottonseed meal. No effect of the level of substitution of sorghum on feed intake was found; however, growth performance decreased when the level of substitution of corn by sorghum increased. Cottonseed meal was substituted to soybean meal up to 40% and found to increase feed intake while reducing growth performance. Young birds were not more sensitive to these ingredients than older birds since there was no negative effect of these ingredients on performance in the starter phase. Results obtained for sorghum pointed out the necessity to find technological improvements that will increase the utilization of these feedstuffs in broiler diet. An additional work is scheduled to validate these statistical results *in vivo* and to evaluate the interactions induced with the simultaneous inclusions of sorghum, millet and cottonseed meal in broiler feeding.

## Implications

As the demand in feed ingredients for poultry production is increasing, finding alternatives to corn and soybean meal utilization becomes a necessity. Conflicting results were reported in literature on the use of sorghum or millet to replace corn, and cottonseed meal as replacement to soybean meal. This study proposed quantitative knowledge of broiler response to dietary inclusion of sorghum, millet and cottonseed meal, which might lead to an increasing utilization of these feedstuffs.

## Introduction

As a consequence of the consistent development of animal production, especially poultry production, there is an increasing demand for feed ingredients supplying energy and protein for poultry (Rae and Ngaya, 2010). Corn and wheat are the major cereal grains used with soybean meal in the least cost feed formulation for poultry (Rae and Ngaya, 2010; Ravindran, 2013a). The gap between local supply and demand for these major feed ingredients is expected to widen over the coming decades (Ravindran, 2013b). A strong increasing trend and a high variation of the prices of cereals grains and soybean meal have been observed in the last years. According to INSEE database, corn prices varied from $0.08 per kg in January 2000 to $0.16 per kg in December 2014, whereas for soybean meal, it varied from $0.18 per kg in January 2000 to $0.37 per kg in December 2014 (INSEE, 2015). The prices volatility and changes in the availability of corn and soybean meal have spurred interest in using other feed ingredients produced in large scale (Ravindran, 2013b).

While sorghum (*Sorghum bicolor*) is the fifth major cereal produced in the world (Heuzé *et al.*, 2012), millet (*Pennisetum glaucum*, *Setaria italica*) has been cultivated worldwide and used in animal nutrition (Heuzé and Tran, 2012). A protein-rich feed, cottonseed meal (*Gossypium spp.*) is a common feedstuff for animals, notably in cotton-producing areas such as India, China and United States (Heuzé *et al.*, 2013). Several studies showed the interest of using sorghum or millet as alternatives to corn, and cottonseed meal in partial replacement of soybean meal. Utilization of millet in broiler feed resulted in similar (Davis et *al.*, 2003; Hidalgo *et al.*, 2004) or improved performance (Baurhoo *et al.*, 2011) compared with corn-based diets, whereas conflicting results were reported for sorghum and cottonseed meal. Some studies showed a reduction in feed intake and weight gain in sorghum-based diets (Jacobs and Parsons, 2013), whereas others indicated similar performance as the control diet (Jacob *et al.*, 1996). According to some studies (Azman and Yilmaz, 2005), cottonseed meal is a suitable replacement for soybean meal, observing that poultry performance was not different between the two ingredients. However, Watkins *et al.* (1993) reported an increased feed intake with cottonseed meal and a higher feed conversion ratio (FCR) compared with soybean meal-based diets.

Based on several studies, there is no consensus on the recommended level of substitution of these ingredients in broiler feeding. Fifty percent corn replacement with low-tannin sorghum was possible for broiler diets, whereas 100% corn substitution had negative effects on the intestinal mucosa and performance (Torres *et al.*, 2013). However, broilers can be fed up to 100% low tannin sorghum-based diets with similar FCR as the control diet (Jacob *et al.*, 1996). Proposed replacement of corn by millet ranged from 10% to 100% (Hidalgo *et al.*, 2004; Goodarzi Boroojeni *et al.*, 2011). According to Baurhoo *et al.* (2011), substitution rates of millet greater than 50% significantly improved BW and FCR compared with a corn-based diet, whereas no difference was reported between 5% and 75% substitution for Davis et *al.* (2003) and Manwar and Mandal (2009). No detrimental effect on broiler performance was pointed out up to 20% soybean meal substitution by cottonseed meal in the diet (Azman and Yilmaz, 2005), whereas performance was sometimes decreased at much lower inclusion rate. Given all these contradictory results on the level of substitution effect, a dose–response impact of these feed ingredients inclusion should be therefore studied.

All the above information demonstrates that there is no clear response of the effects of the use of sorghum, millet and cottonseed meal in poultry diet. Because results from a single classical experiment are specific to conditions under which observations were made, they cannot be the basis for a large inference space (Sauvant *et al.*, 2008). It is therefore useful to collect results from these studies and apply relevant statistical tools to allow drawing objective conclusions. Meta-analysis is a relevant statistical method to aggregate data from previous published research and to quantify knowledge (St-Pierre, 2001; Sauvant *et al.*, 2008). Thus, the objectives of this work were (i) to determine whether the presence of sorghum, millet and cottonseed meal in broiler feeding will affect the performance and (ii) to investigate the quantitative effect of partial or total substitution of corn with sorghum and millet, and partial replacement of soybean meal with cottonseed meal on performance in broiler.

## Material and methods

### Description of the database

Peer-reviewed publications investigating utilization of sorghum, millet and cottonseed meal as partial or total replacement of corn and soybean meal in broiler feeding were selected from 1990 to 2013. The inclusion of these studies was based on three criteria: (i) experiments involving commercial broiler lines; (ii) experiments reporting at least two of these variables: average daily feed intake (ADFI), average daily gain (ADG) or FCR; (iii) experiments detailing ingredients lists and basic nutritional characteristics of experimental diets. Thus, a database containing 190 treatments was established from 17 papers representing 29 experiments. For each experiment, information describing animals (line, sex, number of birds per replicate, age, BW), experimental conditions (birds housing, diet composition) and measured parameters was recorded. In publications where several experiments were reported, each experiment was identified with a separate code. The complete list of references used for the meta-analysis is given in Supplementary material S1.

### Calculations

Treatment average was considered as the experimental unit. Summary statistics of the data used in the study are presented in Table 1. It can be observed that all the control diets were mainly based on corn and soybean meal. Sorghum and millet were substituted to corn and cottonseed meal was used to replace soybean meal. It was observed that in the experiments contained in the database, the tested feed ingredients were included to substitute the control feedstuffs. However, to ensure similar nutrients supply, the experimental diets were formulated with changes made in other feed ingredients inclusion rates. For instance, in millet-based diets, the level of inclusion of soybean meal was reduced compared with the control diet, whereas in cottonseed meal diets, the level of oil in the diet was increased. Therefore, the levels of substitution of sorghum and millet to corn and cottonseed meal to soybean meal were re-calculated according to equation (1):(1)
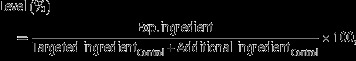
with Exp.ingredient being the inclusion rate of sorghum, millet or cottonseed meal in the experimental diet; Targeted ingredient_control_ the level of inclusion in the control diet of corn or soybean meal; Additional ingredient_control_ the level of inclusion in the control diet of the other feed ingredients modified in the experimental diets.

**Table 1:** Diets nutrients composition and average performance collected in sorghum, millet and cottonseed meal databases used for the meta-analysis (mean±s.e.)

	Database					
	Sorghum		Millet		Cottonseed meal	
	Control	Exp.^1^	Control	Exp.	Control	Exp.
Number of publications	8		5		4	
Number of experiments	12		10		7	
Starter phase	*n*=40		*n*=37		*n*=28	
Ingredient (%)						
Corn	49.2±1.4	29.2±1.1	54.7±1.7	30.8±2.4	53.8±1.0	47.5±0.3
Soybean meal	36.3±0.7	35.4±0.6	35.9±0.9	31.6±0.8	33.8±1.3	17.9±0.8
Oil	4.7±0.2	4.1±0.2	2.5±0.3	3.3±0.2	5.0±0.0	7.5±0.0
Exp. feed ingredient	–	39.2±3.2	–	36.6±3.7	–	19.6±0.7
ME (MJ/kg)^2^	12.78±0.03	12.74±0.04	12.56±0.12	12.46±0.08	13.38±0.02	13.38±0.01
CP (%)^2^	23.1±0.2	22.7±0.1	21.9±0.4	21.6±0.2	22.3±0.3	23.2±0.17
Performance						
Final BW (g)	408±70	458±56	522±34	503±18	732±37	756±12
ADFI (g/bird per day)	35.1±4.0	39.2±3.2	39.0±2.5	39.0±1.6	58.2±2.5	60.3±1.1
ADG (g/bird per day)	22.6±3.2	24.1±2.5	27.4±1.2	27.2±0.7	38.2±2.7	39.4±1.0
FCR	1.68±0.10	1.73±0.08	1.36±0.07	1.33±0.05	1.54±0.08	1.54±0.02
Grower phase	*n*=40		*n*=32		*n*=19	
Ingredient (%)						
Corn	52.1±1.9	19.4±3.1	58.7±2.4	34.8±3.2	63.1±2.9	57.5±2.6
Soybean meal	25.1±1.9	28.5±1.0	31.0±1.8	28.2±1.0	22.2±3.1	15.2±2.3
Oil	3.3±0.8	2.4±0.3	2.7±0.3	3.6±0.2	–	–
Exp. feed ingredient	–	54.5±1.5	–	36.2±4.1		21.3±2.9
ME (MJ/kg) ^2^	14.4±0.4	14.3±0.2	12.83±0.17	12.62±0.10	13.40±0	13.40±0
CP (%)^2^	20.0±0.3	20.8±0.3	20.0±0.6	20.0±0.4	20.2±1.1	20.2±0.7
Performance						
Final BW (g)	1952±275	1700±161	2026±164	2007±106	2663±109	2492±83
ADFI (g/bird per day)	143±15	115±10	144±18	135±9	156±3	155±3
ADG (g/bird per day)	52.8±6.6	47.6±4.3	69.5±5.3	69.8±3.7	75.0±1.8	71.6±1.4
FCR	2.44±0.18	2.36±0.11	2.04±0.14	1.95±0.08	2.16±0.08	2.17±0.06
Reference^3^	Douglas *et al.* (1990), Nyachoti *et al.* (1996), Jacob *et al.* (1996a,b), Ayssiwede *et al.* (2009), Kwari *et al.* (2011), Jacobs and Parsons (2013), Torres *et al.* (2013)		Davis *et al.* (2003), Hidalgo *et al.* (2004), Manwar and Mandal (2009), Baurhoo *et al.* (2011), Goodarzi Boroojeni *et al.* (2011)		Gamboa *et al.* (2001), Henry *et al.* (2001), Sterling *et al.* (2002), Azman and Yilmaz (2005)	

ME=metabolizable energy. Values indicated for diet composition are the average of the amount of ingredients (%) included in experimental treatments. The sum of ingredients is therefore not necessarily 100%.

1 Exp.: Experimental.

2 ME and CP contents are the average reported values in the publications.

3 The complete list of references used for the meta-analysis is given in Supplementary material S1.

**Figure 1:**
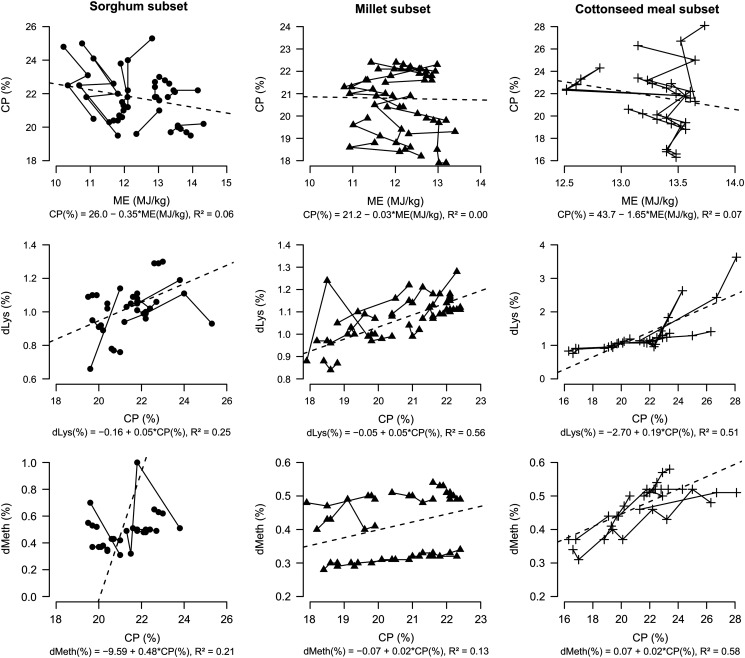
Relationship between the calculated metabolizable energy (ME) and CP contents and CP and amino acids contents of diets used in sorghum (●), millet (▲) and cottonseed meal (+) experiments, respectively. Each point is a treatment average and observations are connected within each experiment. The dashed lines represent the linear adjustment between the two variables.

Information about the feed ingredient cultivar or variety used was rarely mentioned in the publications and not all nutrients contents were given in the publications. Therefore, to ensure consistency within the database, the nutritional values (metabolizable energy, CP and amino acid) of each treatment were estimated using NRC tables of feedstuffs composition (NRC, 1994). *S. bicolor* composition was chosen for treatments involving sorghum, whereas pearl millet (*P. glaucum*) was retained for millet experiments. Nutritional composition of cottonseed meal-based diets was estimated using cottonseed meal (*Gossypium spp.*) prepressed solvent extracted, 44% protein (NRC, 1994). Calculated nutritional composition of the treatments in each experiment is illustrated Figure 1. Each point is a treatment average. Large nutritional changes have been observed between and within experiment. No relationship existed between ME and CP contents. For a similar ME content in cottonseed meal diets, different levels of CP were observed. Lysine and methionine contents were positively related to protein level in the diet.

Production phases were coded according to experimental periods mentioned in the publications. Thus, starter phase covered data collected between 0 and 21 days of age, whereas the growing phase ranged from 21 to 42 days of age. In sorghum-based experiments, a study ranging from 29 to 57 days of age (Jacob *et al.*, 1996) was included in the growing phase. Descriptive statistics showed ADFI, ADG, final BW and FCR varied between each ingredient database (Table 1). Bird responses to an experimental diet were calculated relative to the control as absolute values (Experimental−Control) or as percentages ((Experimental−Control)/Control). These values were then reported as *δ*ADFI, *δ*ADG, *δ*ME intake and *δ*CP intake, for ADFI, ADG, ME intake and CP intake, respectively. *δ*FCR was calculated as absolute values (Experimental−Control) for FCR. The advantage of using *δ* values is to take into account a large part of the variation existing between experiments.

### Statistical analysis

Data analyses were performed using R version 3.0.2 (R Core Team, 2013). The whole database was separated in three subsets of data with sorghum, millet and cottonseed meal experiments, respectively. Global and within-study approaches were applied and discussed according to principles reported by St-Pierre (2001). Differences relative to control diet were submitted to one-way mixed effect model to determine whether the presence of sorghum, millet and cottonseed meal in the diets affected broiler response. *δ*ADFI, *δ*ME intake, *δ*CP intake, *δ*ADG and *δ*FCR were then compared with the reference value (*δ*=0.00) of the control diet using equation (2) in each database. An experiment effect was included in the models as a random effect in order to take into account the sources of variation (bird line, environmental conditions and measurements methods) that may exist between experiments (Sauvant *et al.*, 2008):(2)
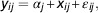
where *y*_*ij*_ is the measured variable (*δ*ADFI, *δ*ME intake, *δ*CP intake, *δ*ADG and *δ*FCR) for treatment *i* (*i*=control, sorghum-, millet- or cottonseed meal-based diets); *j* the experiment number; *α*_*j*_ the random effect for experiment *j*; *x*_*ij*_ regimen effect and *ε*_*ij*_ residual error. Results were considered significantly different if *P<*0.05 and tendencies were noted at *P-*values ⩽0.10.

Another aim of this study was to evaluate the effect of the level of substitution of each ingredient on broiler response. Thus, data of both starter and grower phases were combined to assess this effect on the studied parameters. A two-way mixed effects model (equation (3)), including the production phase effect (starter *v.* grower) was performed on *δ* values expressed as percentage of the control diet for sorghum, millet and cottonseed meal as follows:(3)
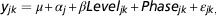
with *y*_*jk*_ being the measured variable in the experiment *j* at the production phase *k*; *μ* the overall intercept with fixed effect; *α*_*j*_ the random effect for experiment *j* on the intercept *μ*; *β*, the coefficient of the level of substitution (*Level*); *Phase* the production phase effect and *ε*_*jk*_ the residual error. Distributions of random effects (*α*_*j*_) and residual error (*ε*_*jk*_) were assumed to be normal. The obtained models were evaluated using different criteria: the significance level of the estimated parameters, the coefficient of determination (*R* ^2^) and the residual variation expressed as root mean square error (r.m.s.e.). The adequacy of the mixed models performed on the response to increasing level of substitution of each ingredient (equation (2)) was also assessed using residuals plots (Observed−Predicted) against predicted values of *Y* to test for linear prediction bias (St-Pierre, 2001).

## Results

### Data consistency

**Figure 2:**
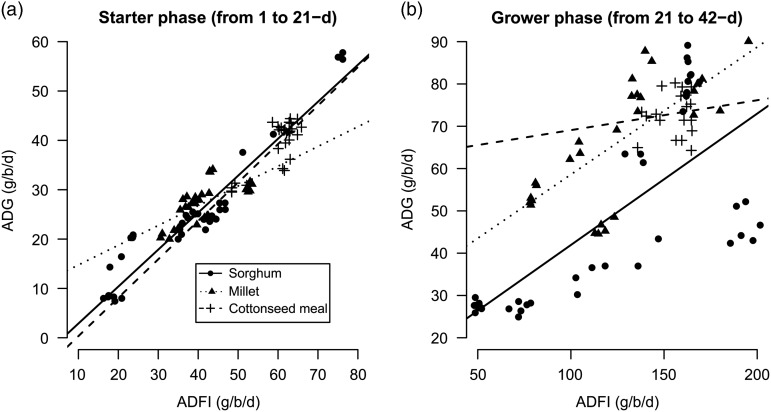
Average daily gain (g/bird per day) as a function of average daily feed intake (g/bird per day) for sorghum, millet and cottonseed meal during starter (a) and grower (b) phases. The lines represent the linear regression between both variables. The overall adjustments for starter phase were: ADG=−4.60+0.75ADFI (*R* ^2^=0.91) for sorghum; ADG=10.75+0.40ADFI (*R* ^2^=0.48) for millet; ADG=−7.45+0.78ADFI (*R* ^2^=0.69) for cottonseed meal. Grower phase. Sorghum: ADG=10.87+0.31ADFI (*R* ^2^=0.52); millet: ADG=28.46+0.30ADFI (*R* ^2^=0.64); cottonseed meal: ADG=62.02+0.07ADFI (*R* ^2^=0.02). Where ADG=average daily gain (g/bird per day), ADFI=average daily feed intake (g/bird per day).

Before any statistical analysis, ADG was expressed as function of ADFI in order to verify the consistency of the database (Figure 2). No clear outliers were denoted in both starter (from 1 to 21 days) and grower (from 21 to 42 days) phases. The results of the regression analysis allowed a conclusion of a relationship between ADFI and ADG, with ADFI explaining 84.3% of ADG variance in starter phase and 54.0% in the grower one. However, for millet-based diets, a lower *R* ^2^ (0.25) was obtained during starter phase (not shown). This was related to one experiment with a much lower FCR (0.85±0.04) compared with what could be expected according to guidelines (1.13). These data were eliminated from other analyses with a new *R* ^2^ of 0.48. In the grower phase, sorghum data could suggest a quadratic model between ADG and ADFI; but the quadratic effect was found to be non-significant in the performed regression test.

Regressions analysis of nutritional composition showed a poor adjustment of calculated nutritional composition to reported composition (results not shown). For ME content in the starter phase, the slope and the intercept were significantly different from 0, meaning that a slight relationship (*R* ^2^=0.31) existed between recalculated and reported ME values. The opposite was observed for the grower phase for ME content with a low *R* ^2^ (*R* ^2^=0.08) and the slope significantly different from 0. CP content was closer to reported data with *R* ^2^=0.17 and 0.56, respectively, for starter and grower phases with a slope different from 0 in both cases. The reason of the differences between diets composition reported in the publications and our own calculations is probably the use of different feed tables, since the composition of ingredients was rarely analyzed by authors and reported in publications. Therefore, to ensure uniformity of information, the nutritional values used in the analyses were those calculated with NRC values (Figure 1).

### Effect of the inclusion of sorghum, millet and cottonseed meal on broiler performance

Differences of performance relative to control diet were shown in terms of absolute values (Experimental−Control) and as percentages relatively to control (Experimental−Control)/Control). No difference was observed in the statistical analyses for absolute values or percentages. Therefore, the results are discussed only as percentage of the control diet (Table 2).

**Table 2:** Responses relative to control diet for feed intake, nutrients intakes and growth performance to sorghum, millet and cottonseed meal utilization

	Sorghum-based diets				Millet-based diets				Cottonseed meal-based diets			
	Mean±s.e. (g/bird per day)	*P* ^1^	Mean±s.e. (% control)	*P*	Mean±s.e. (g/bird per day)	*P*	Mean±s.e. (% control)	*P*	Mean±s.e. (g/bird per day)	*P*	Mean±se (% control)	*P*
*δ*ADFI												
Starter phase	1.23±0.44	**	3.80±1.21	***	−0.60±0.45	ns	−1.75±1.33	ns	1.95±0.38	*	3.31±0.64	*
Grower phase	−6.41±2.54	†	−5.10±1.74	†	−11.37±3.84	†	−5.41±2.00	†	3.75±1.43	*	2.57±3.26	*
*δ*CP intake												
Starter phase	0.23±0.08	**	3.29±0.97	***	−0.15±0.12	ns	−1.96±1.50	ns	1.49±0.21	***	11.60±1.57	***
Grower phase	−1.86±0.58	**	−6.81±1.94	**	−2.20±0.86	ns	−4.12±2.17	ns	2.20±0.38	***	7.41±1.17	***
*δ*ADG												
Starter phase	−0.47±0.31	ns	−1.51±1.33	ns	0.12±0.24	ns	0.43±0.89	ns	−0.14±0.33	ns	−0.12±0.83	ns
Grower phase	−2.31±0.62	**	−5.36±1.42	**	1.93±1.14	ns	2.58±1.57	ns	−2.96±0.38	*	−3.95±1.17	*
*δ*ME intake	(MJ/bird per day)		(% control)		(MJ/bird per day)		(% control)		(MJ/bird per day)		(% control)	
Starter phase	0.02±0.00	***	4.19±1.04	***	−0.03±0.01	***	−7.09±1.61	**	−0.02±0.06	ns	−2.29±0.73	ns
Grower phase	−0.03±0.03	ns	−2.28±1.98	ns	−0.24±0.06	*	−10.57±2.15	**	0.01±0.02	ns	0.64±0.90	ns
*δ*FCR												
Starter phase	0.09±0.02	***			−0.03±0.02	ns			0.05±0.01	ns		
Grower phase	0.01±0.03	ns			−0.15±0.07	ns			0.09±0.05	ns		

ADFI=average daily feed intake; ADG=average daily gain; FCR=feed conversion ratio; ME=metabolizable energy. *δ*ADFI and *δ*ADG are differences relative to control diets expressed in absolute values (Experimental−Control) or as percentages of the control ((Experimental−Control)/Control), respectively, in ADFI and ADG. *δ*FCR was expressed relative to the control in absolute value for FCR. ***P<0.001; **P<0.01; *P<0.05; †*P*⩽0.10. ns=not significant at *P*>0.10.

1 A one-way mixed effect model was performed in each database to determine whether the presence of sorghum, millet and cottonseed meal in the diets affected broiler’s response. *P* is the probability of *δ*ADFI, *δ*ME intake, *δ*CP intake, *δ*ADG and *δ*FCR to be different from the reference value (*δ*=0.00) of the control diet.

#### Starter phase (from 1 to 21 days)

Sorghum-based diets and cottonseed meal-based diets significantly increased ADFI compared with the control diet with about +3.80% and +3.31%, respectively. With millet-based diets, similar feed intake as the control diets was observed (Table 2). ME intake was, however, reduced (−7.09%), whereas the opposite was observed with sorghum-based diets (+4.19%). No difference of ME intake was observed between the control diets and cottonseed meal-based diets. An increase of CP intake was observed when birds were fed with sorghum-based diets and cottonseed meal-based diets in replacement of corn- or soybean meal-based diets, respectively. CP intake was similar among experimental treatments fed millet and those receiving the control diets. No effect of the feed ingredient was observed on growth rate since similar ADG were obtained between control and experimental diets with all three feed ingredients. Birds offered sorghum-based diets increased FCR by 0.09 compared with those fed corn-based diets. Average values obtained for *δ*FCR showed no significant difference between control diet and millet-based diets or cottonseed meal-based diets.

#### Grower phase (from 21 to 42 days)

During the grower phase, differences relative to the control diet in each experiment showed that millet-based diets tended to reduce ADFI (*P=*0.10) but did not impact ADG (Table 2). ADG was found to be lower in sorghum-based diets (−5.36%) compared with the control, whereas a trend was observed for ADFI (*P=*0.05). Birds fed cottonseed meal-based diets increased ADFI by 2.57% while reducing ADG by 3.95% compared with the control. No significant effect of cottonseed meal and sorghum was found on ME intake contrary to millet, which decreased ME intake (−10.57%). CP intake was not affected by millet, but it was affected by sorghum (−6.81%) and cottonseed meal (+7.41%). None of the tested ingredients significantly affected FCR during this phase.

### Broiler response to an increasing level of substitution

**Figure 3:**
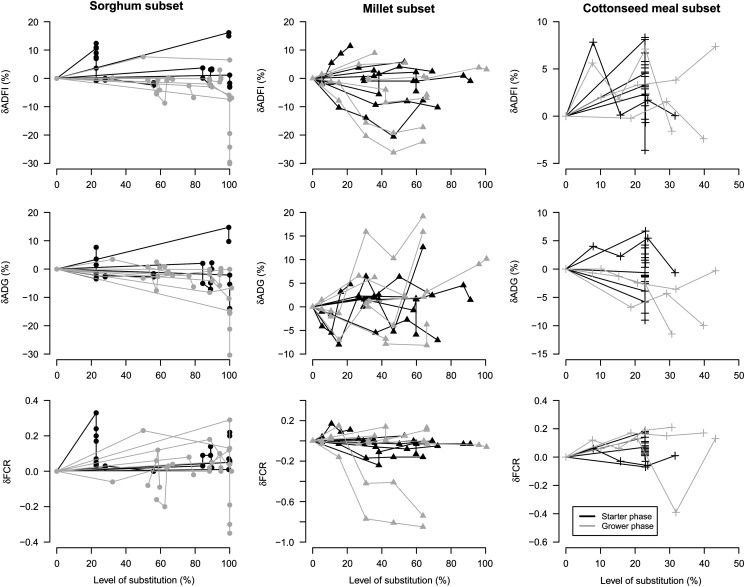
Global and within-study responses of *δ*ADFI, *δ*ADG and *δ*FCR to an increasing level of substitution of sorghum (●), millet (▲) and cottonseed meal (+) for starter phase and grower phase. Observations belonging to one trial are connected with a solid line. ADFI=average daily feed intake; ADG=average daily gain; FCR=feed conversion ratio.

Observed *δ*ADFI, *δ*ADG and *δ*FCR *v*. level of substitution are presented in Figure 3. No linear inter-study effect seems to exist between the level of substitution and any of the performance criteria studied. However, a substantial variation in the response could be observed across trials. Apparently, no difference seems to exist between starter and grower phase for all criteria.

Parameter estimates for equation (2) are reported in Table 3. In sorghum database, no significant effect of the level of substitution was observed on *δ*ADFI and *δ*FCR. However, growth performance consistently decreased when the level of substitution of corn by sorghum increased. The regression analysis also showed that the only major relationship existed between the level of substitution of sorghum and the growth rate (*R* ^2^=0.18). No difference was found for *δ*ADFI and *δ*ADG between the starter and the grower phase except for *δ*FCR, which tended to be higher in the starter than in the grower phase (*P=*0.06).

**Table 3:** Parameter estimates obtained from the mixed effects models (equation(2)) describing the responses in *δ*ADFI, *δ*ADG and *δ*FCR as a function of level of substitution and the production phase for sorghum-, millet- and cottonseed meal-based diets

	Sorghum-based diets			Millet-based diets			Cottonseed meal-based diets		
	Coefficient	s.e.	*P* ^1^	Coefficient	s.e.	*P*	Coefficient	s.e.	*P*
*δ*ADFI (% control)^2^									
Intercept	1.20	2.08	ns	−0.09	2.35	ns	0.60	0.91	ns
Level effect	−0.06	0.03	ns	−0.08	0.04	*	0.06	0.04	*
Phase effect			ns			ns			ns
Level×phase			**			ns			ns
*R* ^2^	0.08			0.03			0.11		
r.m.s.e.	5.66			6.11			2.73		
*δ*ADG (% control)^2^									
Intercept	1.36	1.74	ns	−1.19	1.56	ns	−0.06	1.05	ns
Level effect	−0.07	0.02	***	0.09	0.03	**	−0.16	0.05	*
Phase effect			ns			ns			*
Level×phase			*			ns			ns
*R* ^2^	0.18			0.08			0.09		
r.m.s.e.	4.74			4.83			3.15		
*δ*FCR^3^									
Intercept	0.00	0.03	ns	0.00	0.06	ns	0.02	0.03	ns
Level effect	0.00	0.00	ns	0.00	0.00	ns	0.00	0.00	†
Phase effect			†			ns			ns
Level×Phase			ns			ns			ns
*R* ^2^	0.00			0.05			0.06		
r.m.s.e.	0.10			0.17			0.09		

r.m.s.e.=root mean square error; ADFI=average daily feed intake; ADG=average daily gain; FCR=feed conversion ratio. *R* ^2^: given for the relationship between the variable of interest and the level of substitution. . ***P<0.001; **P<0.01; *P<0.05; †*P*⩽0.10. ns: not significant at *P*>0.10.

1 A two-way mixed effect model including the production phase effect as co-variable and the experiment as random effect was performed on *δ* values from each database to determine the effect of the level of substitution on broiler’s response.

2 *δ*ADFI, *δ*ADG and are differences relative to control diets ((Experimental−Control)/Control), respectively, in ADFI, ADG and FCR.

3 *δ*FCR was expressed relative to the control in absolute value (Experimental−Control) for FCR.

The level of substitution of corn by millet significantly impacted *δ*ADFI, *δ*ADG. A reduction of ADFI was observed with the increasing level of millet in broiler feeding, whereas the opposite was obtained for *δ*ADG. However, the lower *R* ^2^ (*R* ^2^=0.03 and *R* ^2^=0.08, respectively for *δ*ADFI and *δ*ADG) indicated the lack of strong linear relationship between the level of substitution and these two criteria of performance. No influence of the level of substitution of millet on *δ*FCR was observed. These conclusions were similar in starter and grower phases since no effect of the production phase was observed on all the performance criteria studied. For cottonseed meal diets, a significant impact of the level of substitution was obtained on *δ*ADFI and *δ*ADG but it did not influence *δ*FCR. *δ*ADG was affected by the production phase and found significantly higher in the grower phase. Lower values of r.m.s.e. (root mean square error) were observed for all criteria for sorghum, millet and cottonseed meal databases thus leading to a conclusion of a good accuracy of the fitted models.

### Model evaluation

**Figure 4:**
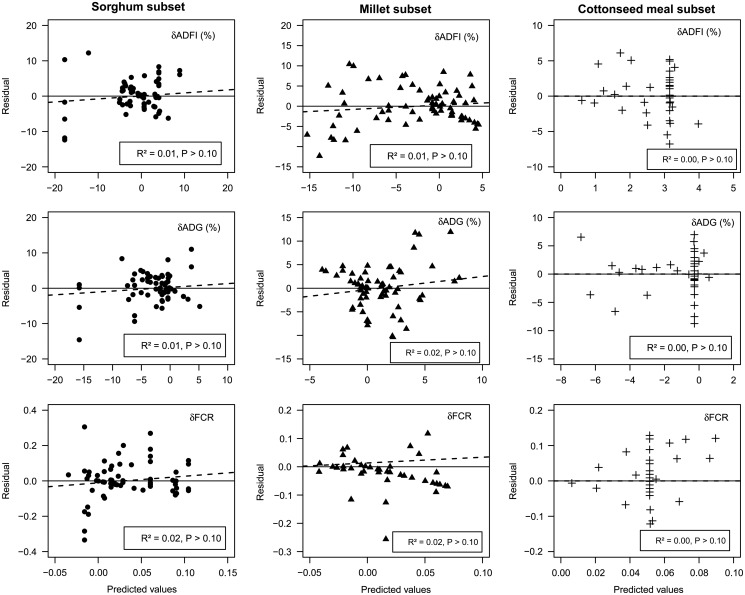
Plots of the Residual (Observed−Predicted) *v.* predicted values of the mixed effects models (equation (2)) for sorghum- (●), millet- (▲) and cottonseed meal- (+) based diets. Dashed lines represent the linear adjustment of residuals to predicted values.

The residuals *v.* predicted values for all mixed models performed on the response to level of substitution are presented in Figure 4. No obvious patterns are evident in the plots. However, the slight deviation from the solid line (Observed=Predicted) observed in sorghum and millet indicated a small difference between observed and predicted values. Better predictions were obtained with cottonseed meal models, since both solid regressions and dashed lines cannot be distinguished. Despite some largest residuals observed in all ingredients, the lack of correlation (*R* ^2^≈0) indicated a fairly good prediction of *δ*ADFI, *δ*ADG and *δ*FCR. Overall, all of the intercepts obtained from the regressions analysis were significantly similar to 0 (*P>*0.10) and the slope, almost null (*P>*0.10), thus confirming the above mentioned results of the level of substitution on performance.

## Discussion

The objective of the current study was to evaluate the effects of partial or total substitution of corn by sorghum and millet and the effect of soybean meal replacement by cottonseed meal on broiler performance. Results obtained indicated that millet can partially or totally replace corn in broiler feeding without any detrimental effect on performance and this is consistent with several authors (Davis *et al.*, 2003; Hidalgo *et al.*, 2004). No significant difference in feed intake and growth performance were observed with millet-based diets when compared with corn-based diets. However, numerical differences were observed with ADFI, which tended to be lower in millet-based diets. Despite the slight reductions of feed intake and ME intake, birds fed millet-based diets have similar growth performance to corn-based diets. This could be related to the well-balanced amino acid profile of pearl millet grains as well as the high essential amino acid concentrations and the high digestibility rate of these amino acids (Adeola and Orban, 1995; Yin *et al.*, 2002).

Sorghum and cottonseed meal affected broiler performance in agreement with previous works (Watkins *et al.*, 1993; Kwari *et al.*, 2011). An increase in ADFI was obtained with cottonseed meal-based diets compared with control ones in both starter and grower phases. Since cottonseed meal-based diets were lower in calculated ME content (*δ*ME=−0.73±0.07 MJ/kg and *δ*ME=−0.25±0.05 MJ/kg, in starter and grower phases, respectively), birds adjust their consumption to satisfy energy requirements (Pérez-Bonilla *et al*., 2012). Conversely, the reduction of ADFI observed with sorghum-based diets in the grower phase was associated with the higher energy level of these diets (*δ*ME=+0.35±0.07 MJ/kg) compared with the control diets (*P<*0.05). During the starter phase, a higher consumption was observed with sorghum-based diets and was associated with the sorghum particle size in these experiments. Whole and coarse ground sorghum was offered to birds and found to increase feed intake in comparison with finer particles (Jacobs and Parsons, 2013). As reported by Nir *et al.* (1990), bird has a preference for larger particles and this preference increases with age.

Growth performance was reduced in sorghum- and cottonseed meal-based diets in the grower phase and this agreed with the reports of Ojewola *et al.* (2006) and Jacobs and Parsons (2013). This might be related to the content of anti-nutritional factors of both sorghum and cottonseed meal. Tannins and phytate in sorghum are known to form complexes with protein and carbohydrates, particularly with starch (Selle *et al.*, 2010), thus leading to a reduction of nitrogen and starch digestibility (Mahmood *et al.*, 2014). In addition, the effect of tannin on bird’s performance depends on its dietary level and the amount of feed ingested. Birds fed high tannin diets suffered from a severe decrease of growth compared with low tannin or control fed birds (Mahmood *et al.*, 2006). In cottonseed meal, free gossypol binds to lysine and reduces the lysine available for absorption (Henry *et al*., 2001). This component also inhibits the activity of pepsin and trypsin in gastro-intestinal tract, thereby reducing the digestibility of protein and growth in broilers (Nagalakshmi *et al.*, 2007). Bird’s tolerance to free gossypol depends on their age, protein content and quality, duration of feeding and presence of minerals especially the iron content in the diet (Nagalakshmi *et al.*, 2007). According to Panigrahi and Morris (1991), significant improvements of feed intake and egg production were obtained when laying hens were given iron treated cottonseed meal-based diets. Therefore, increasing protein or amino acid in the diet was shown to overcome the deleterious effects of tannins and gossypol (Nagalakshmi *et al.*, 2007). Since iron was not included in cottonseed meal-based diets of this meta-analysis, its utilization in broiler diet can be a way to improve the performance. Phytase supplementation can also be suggested to enhance amino acid digestibility in sorghum-based diets (Selle *et al.*, 2010).

Overall, no significant differences were observed regarding the influence of millet-based diets and cottonseed meal-based on *δ*FCR. Though, numerical reductions of FCR were obtained with millet-based diets in agreement with Baurhoo *et al.* (2011) and Goodarzi Boroojeni *et al.* (2011) and in line with the reduced feed intake observed in both starter and grower phases. A greater CP digestibility and the changes in the small intestine mucosa morphology were reported to be the factors leading to a better efficiency with millet utilization (Baurhoo *et al.*, 2011; Goodarzi Boroojeni *et al.*, 2011). On the contrary, feed efficiency was reduced with sorghum-based diets in starter phase accordingly with the higher consumption observed for equivalent growth performance as the corn-based diets.

Only a few differences (*δ*ADG and *δ*FCR) were observed in starter phase compared with the grower phase for sorghum-based diets and cottonseed meal-based diets. There is no evidence of cumulative effect of gossypol or tannins in the literature; therefore, it can be hypothesized that the effect of sorghum and cottonseed meal observed in the grower phase is related to specific conditions (e.g. feed formulation) of the set of experiments selected in this production phase rather than a specific age-dependent effect. The lack of negative effect in starter phase does not justify restricting the use of these ingredients in younger birds.

The fitted models showed a very weak linear relationship between the level of substitution and the investigated criteria of performance. Although a significant effect of the level of substitution on *δ*ADFI and *δ*ADG was obtained with millet- and cottonseed meal-based diets, low *R* ^2^ and slopes were determined. Based on these models, an average substitution of 33% of corn by millet, for example, would result in an increase of ADG of 1.78%, whereas soybean meal replacement by 17% of cottonseed meal would decrease growth performance by 2.78%. It could then be hypothesized that no strong effect of the level of substitution was observed in millet-based diets and cottonseed meal-based diets, consistently with Davis *et al.* (2003) and Manwar and Mandal (2009). However, in sorghum-based diets, a negative correlation (*R* ^2^=0.18) and high *P*-value were observed regarding the effect of the level of substitution of this feed ingredient on growth performance. This is in line with Kwari *et al.* (2011) and Torres *et al.* (2013) who demonstrated that higher substitution levels of corn by sorghum decreased growth performance. These results of performance might be explained by a dose–response effect of the anti-nutritional factors present in sorghum (phytate or tannins). Accumulation of phytate and tannins in the diet could dramatically depress protein digestion and thereby decrease protein synthesis for growth (Selle *et al.*, 2010).

Variability in bird response to utilization of these feed ingredients was observed, suggesting that this is related to some unknown factors not considered in this analysis. Given that environmental conditions affected bird’s response (Syafwan *et al.*, 2012), information about temperature and relative humidity throughout the experiments could enhance the models’ precision. In starter and grower phase, the intake of cottonseed meal-based diets increased with the level of substitution. At the same level of substitution, a wide variability was also observed and related to different experiments (Khalid *et al.*, 2000) involving extruded cottonseed meal supplemented or not with amino acids. This variability in bird response could also be explained to the dietary level of anti-nutritional factors (Mahmood *et al.*, 2006), since no such information was detailed in the selected publications. Furthermore, taking the experiment effect as a fixed effect in a GLM, as suggested by Sauvant *et al.* (2008) in case of heterogeneity between studies resulted in similar results like those obtained with the mixed effect models.

This meta-analytical approach provides significant quantitative knowledge to the utilization of these ingredients in both starter and grower phases. Cottonseed meal-based diets were given upon 40% of substitution and found to increase feed intake while reducing growth performance. Analyses showed that diets containing millet produced similar performance as the corn-based diets, whereas sorghum-based diets decreased growth performance. No major effect of the level of substitution was observed with millet and cottonseed meal; whereas with sorghum-based diets, a negative relationship was pointed out between the level of substitution of sorghum and growth performance. This study highlighted the necessity to find technological improvements that will lead to an increased utilization of these alternative feedstuffs, especially sorghum in poultry. However, in this investigation, information on environmental conditions throughout experiments, feedstuff varieties or anti-nutritional factors contents was not available and therefore these variables as such could not be included as factors in the analysis. This may be considered as a limiting factor to the present study. Thus, in order to evaluate the accuracy of the obtained models, two trials were conducted with sorghum, millet and cottonseed meal. The potential interactions that might be induced with the simultaneous inclusion of these ingredients on broiler performance and nutrients digestibility were also assessed. Results of these trials are presented in a separate paper.

## Supplementary material

For supplementary material accompanying this paper visit http://dx.doi.org/10.1017/S1751731115000282. click here to view supplementary material
