# Supplementary material for: Inclusion of sorghum, millet and cottonseed meal in broiler diets: a meta-analysis of effects on performance
Source: Animal. 2015 Mar 4;9(7):1120–30. doi: 10.1017/S1751731115000282 (PMC4492222; doi:10.1017/S1751731115000282)
Supplement: Supplementary file 1 [file S1751731115000282sup001.docx]

**Supplementary material S1**

The list of references used for the meta-analysis is presented below:

Sorghum

Ayssiwede SB, Azebaze SPA and Missohou A 2009. Essais de substitution du maïs par le sorgho dans la ration: effets sur les performances zootechniques des poulets de chair. Revue Africaine de Santé et de Productions Animales 7, 25-32.

Douglas JH, Sullivan TW, Bond PL, Struwe FJ, Baier JG and Robeson LG 1990. Influence of grinding, rolling, and pelleting on the nutritional value of grain sorghums and yellow corn for broilers. Poultry Science 69, 2150-2156.

Jacob JP, Mitaru BN, Mbugua PN, Blair R 1996a. The effect of substituting Kenyan Serena sorghum for maize in broiler starter diets with different dietary crude protein and methionine levels. Animal Feed Science Technology 61, 27-39.

Jacob JP, Mitaru BN, Mbugua PN, Blair R 1996b. The feeding value of Kenyan sorghum, sunflower seed cake and sesame seed cake for broilers and layers. Animal Feed Science Technology 61, 41-56.

Jacobs C and Parsons CM 2013. The effects of coarse ground corn, whole sorghum, and a prebiotic on growth performance, nutrient digestibility, and cecal microbial populations in broilers fed diets with and without corn distillers dried grains with solubles. Poultry Science 92, 2347–2357.

Kwari ID, Diarra SS, Saleh B, Bovoa PR, Ramat OA and Tochukwu D 2011. Growth, hematology and serology of broiler chickens fed different cultivars of sorghum as replacement for maize in the semi-arid zone of nigeria. International Journal of Poultry Science 10, 608-612.

Nyachoti CM, Atkinson JL and Leeson S 1996. Response of broiler chicks fed a high-tannin sorghum diet. Journal of Applied Poultry Research 5, 239-245.

Torres KAA, Pizauro Jr. JM, Soares CP, Silva TGA, Nogueira WCL, Campos DMB, Furlan RL and Macari M 2013. Effects of corn replacement by sorghum in broiler diets on performance and intestinal mucosa integrity. Poultry Science 92, 1564–1571.

Millet

Baurhoo N, Baurhoo B, Mustafa AF and Zhao X 2011. Comparison of corn-based and Canadian pearl millet-based diets on performance, digestibility, villus morphology, and digestive microbial populations in broiler chickens. Poultry Science 90, 579–586.

Davis AJ, Dale NM and Ferreira FJ 2003. Pearl millet as an alternative feed ingredient in broiler diets. Journal of Applied Poultry Research 12, 137–144.

Hidalgo MA, Davis AJ, Dale NM and Dozier III WA 2004. Use of whole pearl millet in broiler diets. Journal of Applied Poultry Research 13, 229–234.

Goodarzi Boroojeni F, Samie AH, Edriss MA, Khorvash M, Sadeghi G, Van Kessel A and Zentek J 2011. Replacement of corn in the diet of broiler chickens using foxtail millet produced by 2 different cultivation strategies. Poultry Science 90, 2817–2827.

Manwar SJ and Mandal AB 2009. Effect of high moisture storage of pearl millet (*Pennisetum typhoides*) with or without feed enzymes on growth and nutrient utilization in broiler chickens. Animal Science Journal 80, 438–445.

Cottonseed meal

Azman MA and Yilmaz M 2005. The growth performance of broiler chicks fed with diets containing cottonseed meal supplemented with lysine. Revue de Médecine Vétérinaire 156, 104-106.

Gamboa DA, Calhoun MC, Kuhlmann SW, Haq AU and Bailey AC 2001. Use of expander cottonseed meal in broiler Diets formulated on a digestible amino acid basis. Poultry Science 80, 789–794.

Henry MH, Pesti GM, Bakalli R, Lee J, Toledo RT, Eitenmiller RR and Phillips RD 2001. The performance of broiler chicks fed diets containing extruded cottonseed meal supplemented with lysine. Poultry Science 80, 762–768.

Sterling KG, Costa EF, Henry MH, Pesti GM and Bakalli RI 2002. Responses of broiler chickens to cottonseed and soybean meal-based diets at several protein levels. Poultry Science 81, 217–226.
